# Novel Flame-Retardant Wood-Polymer Composites by Using Inorganic Mineral Huntite and Hydromagnesite: An Aspect of Application in Electrical Engineering

**DOI:** 10.3390/ma18112652

**Published:** 2025-06-05

**Authors:** Gül Yılmaz Atay, Jacek Lukasz Wilk-Jakubowski, Valentyna Loboichenko

**Affiliations:** 1 Department of Metallurgical and Materials Engineering Çiğli, Izmir Katip Çelebi University, İzmir 35620, Turkey; hgulyilmaz@gmail.com; 2 Department of Information Systems, Kielce University of Technology, 7 Tysiąclecia Państwa Polskiego Ave., 25-314 Kielce, Poland; 3 Departamento de Ingeniería Energética, Escuela Técnica Superior de Ingeniería, Universidad de Sevilla, Camino de los Descubrimientos s/n, 41092 Sevilla, Spain; vloboichm@gmail.com; 4 Department of Civil Security, Lutsk National Technical University, Lvivska St., 75, 43000 Lutsk, Ukraine

**Keywords:** wood-polymer composites, electrical engineering, huntite-hydromagnesite mineral, flame retardant, mechanical properties, UL 94

## Abstract

In this study, a flame-retardant wood-polymer composite was produced using huntite-hydromagnesite mineral, recognized for its non- flammability properties. In this context, wood-polymer composites were produced with the co-rotating twin-screw extrusion technique, while polypropylene was applied as the composite matrix, medium density fiberboard waste and inorganic huntite-hydromagnesite mineral were used as the reinforcement material. The proportion of wood powder additives was changed to 10% and 20%, and the huntite and hydromagnesite ratio was changed to 30%, 40%, 50% and 60%. Maleic anhydride grafted polypropylene, i.e., MAPP, was applied as a binder at a rate of 3%. Polypropylene, wood fibers, mineral powders, and MAPP blended in the mixer were processed in the extruder and turned into granules. Structural, morphological, thermal, mechanical, and flame-retardant properties of the composites were analyzed using XRD, SEM, FTIR, TGA, tensile testing, and the UL-94 vertical flammability test. Test samples were prepared to evaluate the physical and mechanical properties with a compression molding machine. It was concluded that the composites gained significant flame retardancy with the addition of huntite hydromagnesite. The potential for using this material in various fields and its compliance with the principles of circular economy and the Sustainable Development Goals (SDG 12) were noted.

## 1. Introduction

In practice, wood, which contains cellulose, hemicellulose, and lignin, is a frequently used material in construction as a result of its anisotropic structure. From the point of view of fire protection, the efforts of many scientists around the world are focused on preventing the spread of fire, slowing the combustion process, and conducting fast and effective firefighting [1,2,3], which is often done using nanotechnology and environmental factors [4,5,6,7,8,9].

Many researchers address various aspects of fire protection in their work, which often involves the search for materials with improved fire-resistant properties. Thus, for extinguishing purposes and to prevent fire spread, among others, cellulose-based hybrid hydrogels can serve [10]. Applications in the materials industry include nanocellulose with borates as a material with high fire resistance [11], conductive aerosols and aerosol-assisted vortex rings [12], which finds justification for their use in firefighting, or flexible poly(vinyl chloride) modified with complex of 3-aminotriazoles with zinc phosphate, to increase the safety of the plastic materials applied [13]. Not surprisingly, scientists are focusing on various wood modifications [14,15,16,17,18,19,20]. They focus on studying the physical and mechanical properties of composites, their thermal behavior, flame retardancy and flammability [21,22,23,24,25,26,27], as well as the development of new environmentally friendly flame-extinguishing techniques [28,29,30,31,32,33,34,35,36,37]. An innovative approach to firefighting technology is the acoustic method, which can be used to extinguish flames from materials that are difficult to extinguish by traditional means. The technology is currently being tested in both a confined space and an outdoor environment. The overriding goal is to find out the limiting possibilities of using this technique in the future. The ongoing work focuses on both the application of acoustic waves and the analysis of their effect on the combustion process [38,39,40,41,42,43,44,45,46,47]. This is important because environmentally friendly firefighting techniques are still being sought. If the tests are successful, acoustic technology can be an alternative to traditional fire protection measures in the long run due to the extinguishing capabilities of waves, including their non-invasive nature of operation [48,49,50]. Remote communication techniques can be used to transmit data from inaccessible locations [51,52,53,54,55,56,57,58].

In the context of fire protection, wood-plastic composites represent an interesting research direction that can find many applications. In practice, it is possible to improve the fire-resistant properties of wood by adding suitable components to the composite [59,60,61,62,63,64,65,66,67,68]. In our previous study on the production of environmentally friendly fire-resistant material from waste wood dust [4], the production of the composite obtained by using wood dust and huntite hydromagnesite mineral is explained in detail. Since polymers, like wood, are flammable, there is a practical need to add components to composites to improve their fire-resistant properties. In this study, huntite (Mg_3_Ca(CO_3_)_4_) and hydromagnesite (Mg_4_(OH)_2_(CO_3_)_3_·3H_2_O) minerals were applied to reduce flammability due to their high decomposition temperature. Other benefits of using inorganic huntite-hydromagnesite mineral include low price, no corrosion, low smoke emission, no emission of acid gases, halogen free, and environmentally safe nature [4].

When analyzing the state-of-the-art, most of the previous studies of the authors investigated the fire-resistant properties of polymer composites with mineral additives. In some, the huntite mineral was applied alone, in some, it was used together with other minerals (there was no wood) [69,70], in some studies investigated the fireproof properties of wood composites with huntite additives (nevertheless, there were no polymers) [4,71], and in some of the studies, the general structure and mechanical properties of wood-polymer composites were investigated—different types of wood were applied (but no mineral additives were used) [72]. Therefore, no fireproof properties were investigated (no mineral—no flame retardant).

In the studies conducted by Atay and Çelik, the use of huntite and hydromagnesite minerals as fire retardants in plastic materials to increase the fire resistance of polymeric materials was investigated in 2010 and 2013 [69,70]. The use of huntite-hydromagnesite mineral as a fire retardant in wood composites to improve the fire resistance was the subject of research described in the article written by Atay in 2021 [4]. In contrast, a study on the effect of the presence of calcite and huntite-hydromagnesite minerals together on the fire resistance and mechanical properties of wood composites was conducted by Atay et al. in 2024 [71]. In the study conducted by Atay and Türkmen [72], while the use of hornbeam, pine and medium density fiberboard waste (as wood material) in wood-polymer composites for construction elements was discussed.

In summary, considering polymers and minerals, there are studies of fireproof polymer composites. When we take into account wood and mineral, there are also fireproof wood composite studies. But when we analyze polymer, wood and mineral together, there is no fireproof wood polymer composite study with mineral huntite-hydromagnesite. Therefore, there was a need to fill the research and literature gaps in this area.

Wood-plastic composite materials are a type of composites obtained by combining wood-based elements with polymers. The use of waste wood dust, in particular, has led to the fact that these composites make a significant contribution to the recycling sector. However, considering that both wood and polymers are easily flammable products, the importance of making these composites resistant to fire becomes evident. The production of non-flammable wood-polymer composites will both increase the areas of use of these materials and make them safer (such composites may find applications in electrical engineering, among others). In our previous studies, we used huntite and hydromagnesite minerals (i.e., huntite-hydromagnesite mineral) to provide fire resistance to polymer materials, and we achieved very successful results. This study involved the development of a flame-retardant wood-polymer composite utilizing huntite-hydromagnesite mineral, which is also known for its non-flammability. In practice, the purpose is to provide flame-retardant properties to wood-polymer composites using a huntite-hydromagnesite mineral. Since there is no such study, this constitutes a scientific novelty. The results obtained were analyzed to determine the structural and morphological properties of the composites. For this purpose, several techniques were applied: X-ray diffraction (XRD), scanning electron microscope (SEM), and Fourier-transform infrared spectroscopy (FTIR). Thermal properties were determined according to thermogravimetric analysis (TGA). Tensile tests were performed to obtain the mechanical properties. Flame-retardant performance was evaluated according to the UL 94 vertical flammability test, contributing to state-of-the-art research on new flame-retardant wood-polymer composites through the use of the inorganic mineral huntite and hydromagnesite.

## 2. Materials and Methods

### 2.1. Materials

In the work, the polymer polypropylene (C_3_H_6_)n (PP)—Petoplen EH 251 (PETKİM Petrochemical Holding A.Ş., İzmir, Turkey) was used as a matrix. Such a matrix provides resistance to environmental factors (moisture, water) and ensures flexibility of the entire wood-polymer composite. The advantages of polypropylene include its low cost, easy fluidity when melted (Tmelting = (160–165) °C), resistance to loads and high impact resistance.

Wood sawdust was waste from furniture production (pine, hornbeam and medium density fiberboard (MDF) from the STARWOOD market) (OSABYA Design Co., İzmir, Turkey) and was used as a reinforcing additive in the wood-polymer composite. To bind hydrophilic wood fibers with a hydrophobic polymer, maleic anhydride-grafted polypropylene (MAPP)—Bondyram 1001 (RESINEX BMY AS, İzmir, Turkey) was used.

The natural mineral huntite hydromagnesite was obtained from the Isparta region (Turkey) and then ground to a powder state (grain size—10 microns) in a ball mill, homogenization of the composition was ensured by sifting and mixing.

### 2.2. Compounding

For the preparation of the wood-polymer compounds, the polypropylene, mineral powders, and sawdust quantities were determined. Based on this estimate, different amounts of powders of huntite-hydromagnesite mineral and sawdust mixtures were prepared (see Table 1, where “Polymer” is PP, “Wood” is sawdust, “Huntite hydromagnesite” is the mineral huntite hydromagnesite). It is worth mentioning that we used one type of wood material, i.e., MDF. In this context, wood powder and sawdust, referring to MDF in this study.

As can be seen in Table 1, there is a 100% PP sample containing only organic polypropylene (this sample has the reference character), while in the other cases the samples also contain various proportions of wood and the inorganic minerals huntite and hydromagnesite in different configurations. The reason for choosing different proportions in the composition of the samples is that it was foreseen that they would affect both the fire resistance and the mechanical properties. The first step is the preparation of raw materials containing polymer, wood, as well as huntite-hydromagnesite mineral to give the samples the appropriate mechanical strength and fire resistance characteristics. MAPP was used as a binder at a rate of 3%. To obtain a homogeneous mixture, the required quantities of PP, wood chips, mineral powder huntit hydromagnesite (Table 1) and MAPP were separately placed in a mixer (LabTech Engineering Company Ltd., Phraeksa, Thailand) for 5 min. The prepared mixture was then placed in the hopper of a twin-screw extruder with a screw diameter of 20 mm (L/D ratio 32:1) (LabTech Engineering Company Ltd.). The composite (PP/sawdust/mineral) was obtained under the following conditions:

- Temperature of the extruder zones—(170–185) °C;
- Screw rotation speed—190 rpm;
- Pressure—10 bar.

The resulting liquid composite, after passing through the extruder die, was cooled with water in a cooling bath and then granulated in a granulator. Sample plates for the studies were obtained using a hydraulic laboratory press (LabTech Engineering Company Ltd.). The composite granules were placed in a mold cavity (size—15 cm × 15 cm) in a press preheated to the operating temperature (200 °C). To prevent direct contact of the composite with the hot press, the mold was placed between Teflon sheets. The pressing of the samples (Table 1) occurred for 20 s in the hot part of the press and for 2 min in the cold part under a pressure of 40 bar.

### 2.3. Characterization

The samples produced and then dried were subjected to multifaceted analysis for the effect of individual components on the mechanical and fire retardant properties of a specific sample (mechanical and fire resistance). The obtained samples were examined to determine their structural and morphological, mechanical and thermal properties, as well as their flame-resistant performance. To determine the phase composition of the material and analyze the crystallographic orientation of the particles, X-ray diffraction analysis of the samples was performed on a Bruker D2 Phaser X-ray diffractometer (Bruker, Billerica, MA, USA), with Ni-filtered Cu-K alpha radiation (k = 1.54 Å). Fourier Transform Infrared Spectroscopy (FTIR) was performed using a Bruker Alpha II (Ettlingen, Germany) spectrometer to identify the functional groups present in the samples. The analysis was carried out in the wavenumber range of 4000–500 cm^−1^ with a spectral resolution of 4 cm^−1^. The mechanical properties were investigated using a SHIMADZU AGS-X 5 kN machine (Shimadzu Corporation, Kyoto, Japan). The specimens were prepared according to ASTM D638 [73] using a mold that cut the plate with blades (tests were carried out at room temperature, with a crosshead speed of 50 mm/min). According to this standard, specimen is a composite in the shape of a dumbbell with a total length for 165 mm, a thickness of 3.2 mm and a gauge length of 50 mm. A scanning electron microscope, Carl Zeiss Sigma 300 VP (ZEISS Sigma, Oberkochen, Germany), was used to obtain the morphological images of composites. The samples were gold plated using the QUORUM Q150 RES device (Quorum Technologies Ltd., Lewes, UK), and the images and the accelerated voltage were set to 5 kV and images were taken at different magnifications. Thermal gravimetric analysis (TGA) of flame retardant composite pellets was performed using a Perkin Elmer STA 8000 device (PerkinElmer, Inc., Shelton, CT, USA) to notice the decomposition and phase formation. The analysis was carried out under nitrogen gas by heating at a rate of 10 °C per minute from room temperature to 900 °C. Flame retardant property tests were performed with the ZLT-ZYS Needle Flame Tester machine (Zhilitong Electromechanical Co., Ltd., Guangzhou, China) according to the UL 94 standard [74]. This test is a standardized method for determining the flammability of materials. The test involves exposing a material sample to flame and observing the reaction that occurs. Accordingly, the flame is transmitted to the material from a certain angle and distance. The flame extinguishing time and dripping ability are measured. The ratings and definitions according to the standard are shown in Table 2 [74].

## 3. Results and Discussion

**Figure 1:**
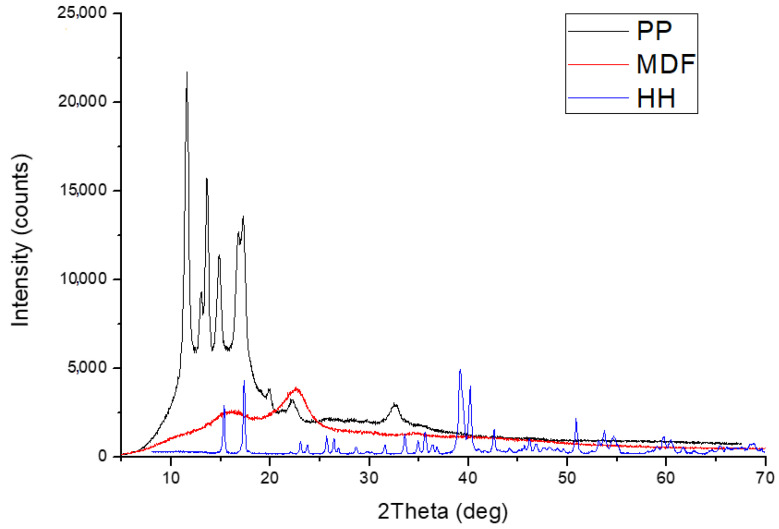
XRD analysis of PP, sawdust (MDF), and huntite hydromagnesite (HH).

To study the crystal structure of materials X-ray diffraction was applied. Figure 1 shows the XRD results for the three composite components, that is, polypropylene, medium density fiberboard waste, and mineral huntite-hydromagnesite. In MDF analysis, the prominent peaks at 22.5° and 18.5° at 2 Theta represent hydrogen bonded cellulose layers corresponding to (101) and (002) planes. In PP analysis, the peaks at 13.8°, 16.6° and 18.3° represent PP crystals corresponding to (110), (040) and (130) diffraction planes. In huntite-hydromagnesite analysis, the peaks at 15.1°, 17.1°, 38.6°, 40.7° and 51.8° represent characteristic peaks of these minerals. The combination of these materials makes it possible to take advantage of both the amorphous properties (including flexibility and moldability) and the crystalline properties (i.e., flame resistance) and mechanical properties (e.g., elastic modulus and stress) of the composite’s additives, which is particularly important for practical applications of the materials.

As can be seen in Figure 1, clear crystalline peaks are observed when using polypropylene, indicating the ordered structure of the molecules of this polymer at certain angles. In turn, broad bands may indicate an amorphous part of the material’s structure. The results obtained from the XRD analysis are characteristic of polymeric materials with limited crystallinity. In the medium density fiberboard waste application, wide scattering bands become apparent, influenced by the structure of the material, which in this case is not ordered (amorphous material). Compared to polypropylene, small peaks are observed because of the partial presence of ordered constituent structures—i.e., MDF. Huntite hydromagnesite is a highly crystalline material, as exemplified by the sharp and distinct peaks in Figure 1, which confirm the presence of stable crystalline regions and the purity of the applied mineral. When X-rays fall on a linear crystal grid, they are scattered in certain directions and form a pattern called a diffraction pattern, which can be used to read information about the structure, size, and direction of crystals in materials under study. From a practical perspective, the narrow scattering band indicates the absence of amorphous regions in the analyzed material.

**Figure 2:**
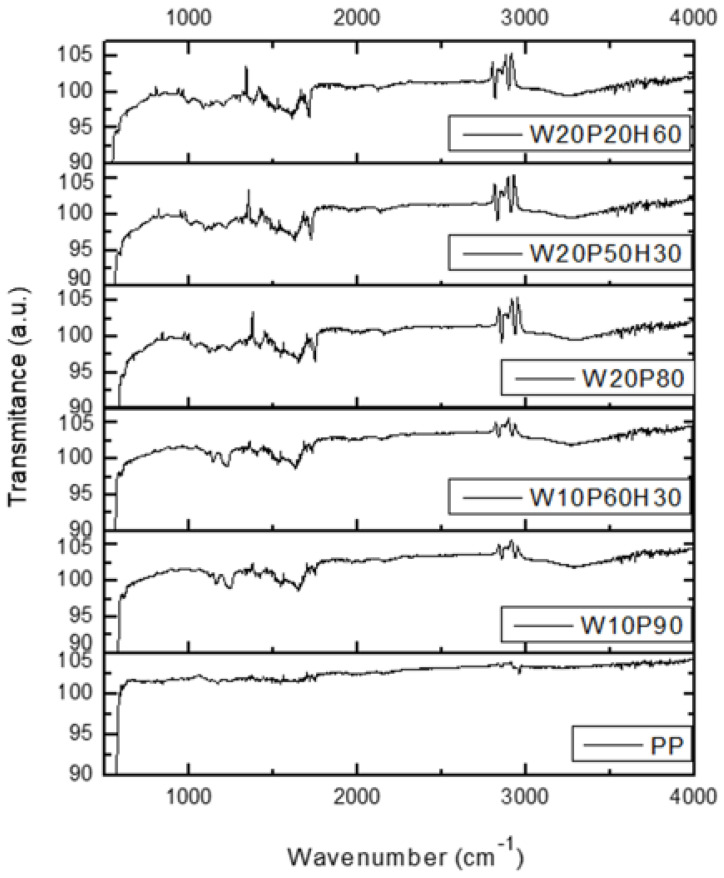
FTIR analysis results of the composites.

The Fourier-transform infrared spectroscopy analyses results are demonstrated in Figure 2. In the case of polypropylene, a strong band was recorded in the range of 2800–3000 cm^−1^, which is characteristic of polyolefins. In turn, weak absorption was observed in the 1500–1700 cm^−1^ range due to the absence of strong functional groups. The addition of wood resulted in the visibility of bands in the 3200–3600 cm^−1^ and 1000–1200 cm^−1^ ranges as a result of the bonding that occurred in the analyzed samples. The FTIR working principle is based on the principle of measuring the vibration of organic bonds in the material by the absorption of infrared (IR) radiation. It is seen that the biggest difference is obtained in the PP and wood dust added samples. Because the peaks that are not in PP have emerged with the addition of wood dust. In the spectra of the samples added with huntite and hydromagnesite minerals, no change was observed in the locations of the peaks. This shows that the mineral powders do not have any effect on the bonds in the PP and wood dust composites.

In practice, poor compatibility of polypropylene with wood is noted because of the hydrophobic (lacking strong functional groups) and hydrophilic properties of the components, respectively. For this reason, as previously indicated, a MAPP compatibilizer was added to improve adhesion processes. The chemical interactions that occur in the composite are also affected by the addition of huntite and hydromagnesite. Clearly visible in the FTIR spectrum in Figure 2, the 1400–1500 cm^−1^ bands confirm the presence of the huntite-hydromagnesite mineral in the analyzed samples, allowing its use with fire-resistant properties for practical applications. On the other hand, one should keep in mind the compatibility with polypropylene, which affects the mechanical properties of the material. The tensile stress, strain, and elastic modulus of the samples are listed in Table 3. Three samples of each different ratio were tested.

**Figure 3:**
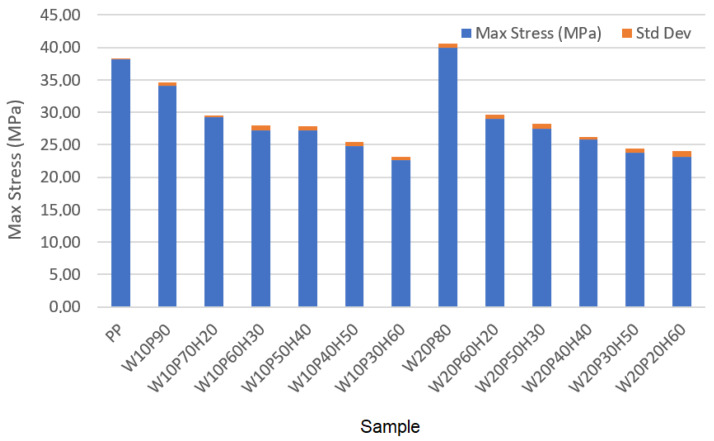
Comparison of maximum stress across samples.

**Figure 4:**
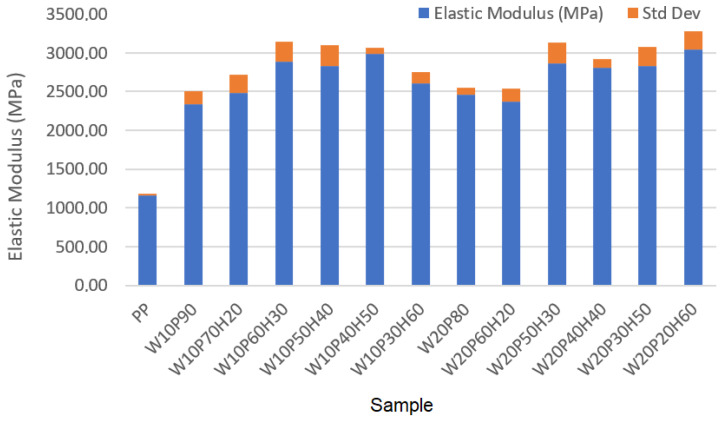
Comparison of elastic modulus across samples.

**Figure 5:**
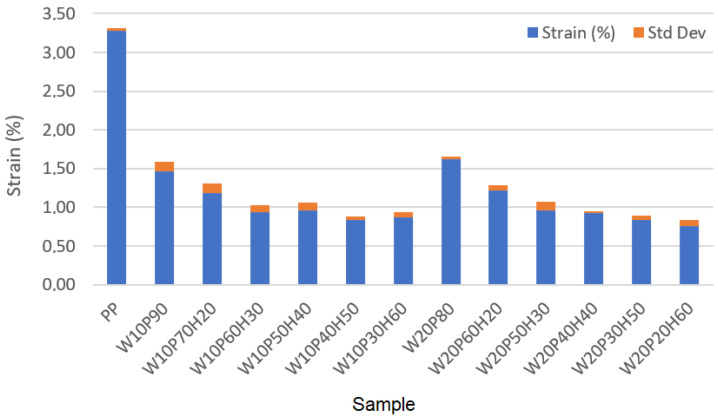
Comparison of strain across samples.

Based on the data presented in Table 3, it can be seen that the addition of wood, as well as huntite and hydromagnesite, to the sample influences its maximum stress, strain, and elastic modulus. These are engineering stress, engineering strain and the elastic modulus calculated accordingly. Additionally, standard deviation values calculated based on each measurement made three times are also shown in Table 3. Pure polypropylene has the highest strain (3.28%), but at the same time the lowest elastic modulus (1163.41 MPa) and one of the highest maximum stresses (i.e., second highest result) at 38.16 MPa were recorded. Each increase in the content of huntite and hydromagnesite contributes to an increase in elastic modulus, but a decrease in the values of maximum stress and strain, which in turn is an undesirable phenomenon. In practice, the highest elastic modulus is recorded for samples with the highest amount of huntite and hydromagnesite in the composition. For the sample W20P20H60 it is equal to 3040.79 MPa, however, its strain is then the lowest at 0.76%. In samples containing 50–60% huntite and hydromagnesite, very low strain values (minimum value equal to 0.76% in analyzed samples) were recorded, although the elastic modulus varied depending on the other components of the samples (W10P40H50, W10P30H60, W20P30H50, W20P20H60). For a small mineral content (10–20%), a good mechanical balance with deterioration of the elastic modulus was observed. A fundamental compromise was obtained between maximum stress and strain for the sample of W20P60H20 (28.96 MPa, 1.22%). The addition of huntite and hydromagnesite to the composities increased its elastic modulus, while lowering its strain capacity. Since increasing the content of huntite and hydromagnesite decreases the mechanical strength of the sample, the best mechanical properties are recorded in samples with less HH and a higher percentage of polymer. To visualize the results, the summary bar graphs obtained for all samples are shown in Figure 3, Figure 4 and Figure 5. In practice, Figure 3 presents the maximum stress comparison across samples, Figure 4 shows the elastic modulus comparison across samples, and Figure 5 illustrates the strain comparison across these samples. As can be seen in Table 3, the deviations of the measurements do not significantly affect the results of the measurements.

The analysis of the results presented above allows one to see some regularities that allow us to assess the applicability of the samples in various fields. For the creation of flexible and strong materials (such as engineering plastics), the PP sample (38.16 MPa, 3.28%) will perform well with high maximum stress and strain, while the material is then flammable. A better compromise between maximum stress and elastic modulus was noted for the W20P80 sample. In this case, the maximum stress increased by 1.73 MPa (i.e., to a value of 39.89 MPa) compared to a sample containing only polypropylene. The strain then decreased by 1.66%, with the elastic modulus increasing by 1298.94 MPa, i.e., to a value of 2462.35 MPa. A good compromise between mechanical resistance and elastic modulus, in turn, is represented by samples containing 20–30% huntite and hydromagnesite. A good balance between elastic modulus and mechanical resistance was noted for the following samples: W10P70H20 (29.24 MPa, 1.18%, 2477.97 MPa) and W20P60H20 (28.96 MPa, 1.22%, 2373.77 MPa). Although mineral huntite-hydromagnesite causes deterioration in the mechanical strength of the material, as its structure becomes more brittle, its applications include areas exposed to high temperatures or fire hazards. In practice, the greater the material’s processability, the better. For electrical engineering applications, taking into account increased fire resistance and elastic modulus (e.g., insulation materials), samples W20P20H60 (23.11 MPa, 0.76%, 3040.79 MPa) and W10P30H60 (22.65 MPa, 0.87%, 2603.45 MPa) appear to be suitable. They are characterized by good fire resistant properties and maximum elastic modulus (the drawback is then lower strain). In terms of the balance between mechanical and fire resistance, it seems to occur in samples with 20–30% HH, where as a result of the use of huntite and hydromagnesite, fire resistance improves, but the sample is still relatively durable and resistant to maximum stress. Increasing the percentage of HH to 40–60% results in an improvement in fire resistance properties that comes at the expense of a reduction in the mechanical strength of the sample. For the reasons, depending on the intended use of the material and the need to ensure the required fire resistance or mechanical strength, the optimal composition of the sample is an appropriate balance of the sample composition.

**Figure 6:**
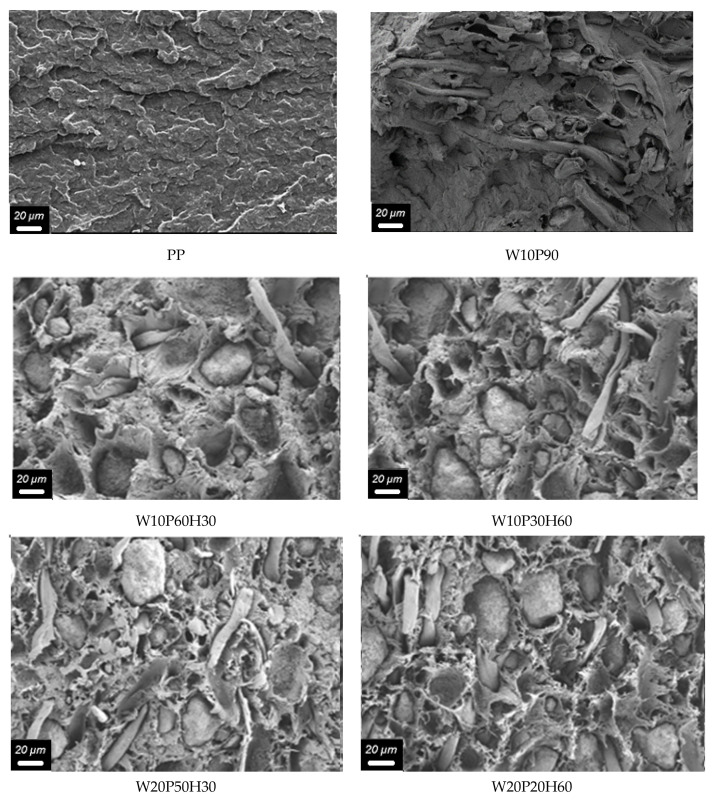
SEM analysis results of composite fracture zones after mechanical test.

Below, in Figure 6 are the results of the SEM analysis results of composite fracture zones after the mechanical test. As can be seen from the presented images, the addition of wood to the polymer matrix worsens the homogeneity of the matrix, promotes the creation of cavities (W10P90) and will probably worsen the mechanical characteristics of the material. At the same time, the addition of the mineral enlarges the cavities (W20P50H30, W10P60H30) and helps to reduce their number (W10P30H60, W20P20H60) by filling these cavities. The presence of cavities, on the one hand, can contribute to their filling with water during operation and the development of microorganisms, on the other hand, the possibility of filling them with a fire-resistant mineral (huntite hydromagnesite) will improve the fire resistance of the material.

**Figure 7:**
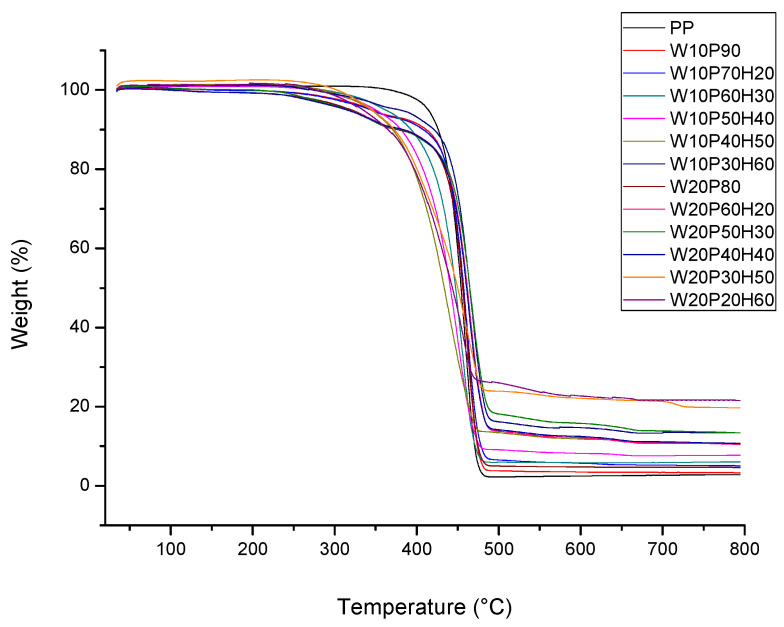
TGA analysis results of the composites.

The thermal properties of the composites were determined according to thermogravimetric analysis and presented in Figure 7. As can be seen, while the temperature increases, a decrease in the weight of the sample is recorded, allowing identification of the main stages of thermal decomposition. If the sample contains low-boiling compounds, depending on the composition—especially MDF, there is a slight weight loss at the initial stage (about 100–200 °C). The pure polymer decomposes almost completely at about 450 °C. Therefore, it is assumed that at a temperature of about 250–400 °C the main decomposition of the polymer and wood takes place. In turn, polypropylene degrades at a slightly higher temperature than wood, i.e., 350–450 °C. As a consequence of this, an earlier and faster weight loss (at about 280–380 °C) is recorded for the samples containing more wood. Since the inorganic huntite-hydromagnesite mineral decomposes at the highest temperature (about 400–700 °C), it provides a kind of thermal barrier. This is because samples with a high content of huntite hydromagnesite show slower weight loss at higher temperatures. Not insignificant is the fact that the heat of the fire causes the decomposition of huntite and the release of CO_2_ into the flames, which contributes to slowing the spread of the fire. Since CO_2_ absorbs heat, there is a beneficial phenomenon of cooling the burning material. In turn, the release of water by hydromagnesite, as well as CO_2_, contributes to reducing the flammability of the composites. In practice, huntite is used mostly as a natural blend with hydromagnesite, resulting in a flame-retardant additive for polymers. In summary, based on Figure 7, it can be concluded that the addition of mineral huntite-hydromagnesite to the sample allows the delay in further weight loss of the sample.

The results of thermogravimetric analysis unequivocally confirmed that the addition of an inorganic mineral huntite-hydromagnesite effectively improves the thermal stability of the material, making the samples more resistant to high temperatures, and showing a wider range of applications for the aforementioned electrical engineering, among others. The more minerals in the sample, the better resistance to the temperature and the lower the weight loss, resulting in greater thermal stability (the samples of W10P30H60 and W20P20H60). This means that samples containing 50–60% huntite hydromagnesite show the slowest weight loss and are more resistant to high temperatures. In practice, however, it all depends on the application. Where it is important to ensure a balance between thermal stability and mechanical strength, samples containing 20–30% huntite hydromagnesite can also be applied (W10P70H20, W10P60H30, W20P60H20, W20P50H30).

The results in terms of evaluating the flame-retardant performance of the composites are given in Table 4. In practice, this performance was evaluated according to the UL 94 flammability test. This test is closely related to the safety of electrical and electronic equipment, since the fire resistance of materials that can potentially be used in electronic and electrical equipment, as well as, for example, in civil engineering. In practice, applications requiring superior fire resistance include materials that can find applications in various areas of the electrical engineering industry, for example, in electrical and electronic equipment housings, for the construction of cable sheaths, wires, or insulating components, or in circuit boards, when fire protection is a key aspect. Since many components made of wood or polymer composites (e.g., equipment housings) are applied in electrical engineering, the use of materials containing the inorganic mineral huntite-hydromagnesite can significantly reduce the risk of fire in areas where electrical installations are located. The use of heat- and fire-resistant as well as lightweight composites in renewable energy could also be a novel application. This is important because many electrical and electronic components, such as equipment housings, wire insulation, circuit boards, and electrical outlets, must meet certain standards to prevent the possible spread of fire. For this purpose, among others, the UL 94 flame-retardant test experiment mentioned below is applied.

Pure polyproplene and samples with a low content of huntite hydromagnesite (≤30%) do not meet the requirements of the UL 94 test (this applies to the samples: PP, W10P90, W10P70H20, W10P60H30, W20P80, W20P60H20). Since the samples burned for a long time after the flame was removed (30 s), their use in applications involving high fire resistance is not recommended. Samples containing 30–40% huntite hydromagnesite in their composition achieved a V-1 rating, meaning that these samples had limited combustion but did not extinguish immediately (this applies to the samples: W10P50H40, W20P50H30, and W20P40H40). The recorded burning time after the flame was removed for these samples was in the range of 35–38 s, which means that these composites effectively limited the burning process by inhibiting the spread of the flame. During these experiments, the highest UL 94 rating of the test, i.e., V-0, was recorded for samples containing 50–60% huntite hydromagnesite (this applies to the samples: W10P40H50, W10P30H60, W20P30H50, and W20P20H60). The burning time after the flame was removed then oscillated between 3–19 s, which means that these samples have very high fire resistance. The shortest burning time of only 3 s was recorded for the W20P20H60 sample, confirming that composites containing high proportions of huntite hydromagnesite are the most effective in providing successful fire protection.

In light of all these results, it has been shown that the use of huntite and hydromagnesite reinforcement in polymer-wood composites has provided a significant gain in terms of increasing fire resistance, which is the main purpose of this study. On the other hand, it has been determined that the mechanical properties have a reconstructive function depending on the use of wood dust in the composites. Walker [75] mentioned in his study that wood is a lignocellulosic material consisting of three main components (cellulose: 42–44%, hemicelluloses: 27–28% and lignin: 24–28%) and some minor components (extractives: 3–4%). The majority of wood is crystalline cellulose. The aligned fibril structure and strong hydrogen bonding of cellulose have high hardness, therefore the addition of wood flour can increase the hardness of thermoplastic-based composites. Lignin, an amorphous polymer, does not contribute greatly to the mechanical properties of wood flour, but it plays an important role in binding cellulose fibrils, which provides effective stress transfer to cellulose molecules. Therefore, wood filler increases the hardness of PP without excessively increasing the density. Wang et al. [76] stated that the effect of incorporating inorganic mineral powders into the wood matrix is to densify and stiffen the cell walls by filling the voids of the cell walls with relatively hard inorganic particles, thus increasing the resistance of the cell walls to deformation and collapse in compression.

When looking at composite structures, it was observed that the incompatibility problem with the matrix caused by the scaly structure of huntite and hydromagnesite was eliminated with wood powder and a more compatible structure was obtained. This explains the improvement in mechanical properties. In addition, it would be appropriate to use different binders in future studies to increase the interaction of wood powders with plastic and mineral powders. In the study conducted by Adhikary et al. [77], it was observed that the addition of a binder improved the compatibility between the wood filler and PP through esterification. Future research is expected to continue exploring the utilization of recycled materials in wood–polypropylene composites, focusing on internal structure, particle characteristics, physico-mechanical performance, fire retardant chemistry, and advancements in biodegradable flame-retardant nano-biocomposites [78,79,80,81].

## 4. Conclusions

In this study, the flame-retardant performance of wood-polymer composites containing huntite hydromagnesite was evaluated, demonstrating that higher proportions of this mineral significantly enhance fire resistance. In addition, samples with a high content of huntite hydromagnesite exhibited slower mass loss at elevated temperatures, confirming their effectiveness in thermal stability. The composites were manufactured using the co-rotating twin-screw extrusion method, with polypropylene serving as the matrix material, while medium density fiberboard and the inorganic huntite-hydromagnesite mineral acted as reinforcement components.

It was found that the addition of the mineral huntite-hydromagnesite causes a deterioration in the mechanical strength of the polymer-wood-mineral composite due to an increase in its brittleness. At the same time, the fire resistance of such materials allows us to talk about the possibility of their use in areas where they are exposed to high temperatures or there is a high fire hazard (insulation materials in the power industry, structures using flammable materials, etc.). In terms of balance between mechanical and fire resistance, this is the optimal sample of polymer-wood-mineral with a mineral content of 20–30%. At the same time, depending on the scope of application and the required characteristics of the material, this ratio may vary.

In the long term, novel flame-retardant wood-polymer composites by using inorganic mineral huntite-hydromagnesite composites may contribute to improving the safety and durability of various electrotechnical systems due to their resistance to high temperatures and fire.

Improving the fire-resistant characteristics of the composite by introducing natural minerals into its matrix confirms the results obtained earlier in this direction and indicates the prospects for using these materials in various fields. This trend also corresponds to the principles of the circular economy (due to the secondary use of wood waste), contributes to the greening of the economy (by using natural materials—wood and natural minerals), and, ultimately, to the achievement of sustainable development goals, and, in particular, SDG 12—Responsible consumption and production.

## Figures and Tables

**Table 1:** The codes and the contents of the composite samples.

No.	Sample Name	Description	PP (%)	Wood (%)	Huntite Hydromagnesite (%)
1	PP		100	–	–
2	W10P90	10% Wood-90% Polymer	90	10	–
3	W10P70H20	10% Wood-70% Polymer-20% Huntite hydromagnesite	70	10	20
4	W10P60H30	10% Wood-60% Polymer-30% Huntite hydromagnesite	60	10	30
5	W10P50H40	10% Wood-50% Polymer-40% Huntite hydromagnesite	50	10	40
6	W10P40H50	10% Wood-40% Polymer-50% Huntite hydromagnesite	40	10	50
7	W10P30H60	10% Wood-30% Polymer-60% Huntite hydromagnesite	30	10	60
8	W20P80	20% Wood-80% Polymer	80	20	–
9	W20P60H20	20% Wood-60% Polymer-20% Huntite hydromagnesite	60	20	20
10	W20P50H30	20% Wood-50% Polymer-30% Huntite hydromagnesite	50	20	30
11	W20P40H40	20% Wood-40% Polymer-40% Huntite hydromagnesite	40	20	40
12	W20P30H50	20% Wood-30% Polymer-50% Huntite hydromagnesite	30	20	50
13	W20P20H60	20% Wood-20% Polymer-60% Huntite hydromagnesite	20	20	60

**Table 2:** UL 94 rating and definitions [74].

UL94 Rating	Definition of Rating
HB	Slow burning on a horizontal part.
V-2	Burning stops within 30 s on a part allowing for drops of vertical flammable plastic.
V-1	Burning stops within 30 s on a vertical part allowing for drops plastic that are not inflames.
V-0	Burning stops within 10 s on a vertical part allowing for drops plastic that are not inflames.
5VB	Burning stops within 60 s on a vertical part allowing for drops plastic that are not inflames, plaque specimens may develop a hole.
5VA	Burning stops within 30 s on a vertical part allowing for drops plastic that are not inflames, plaque specimens may not develop a hole.

**Table 3:** Mechanical test result of wood-polymer composites.

No.	Sample Name	Max Stress (MPa)	*Std. Dev.*	Strain (%)	*Std. Dev.*	Elastic Modulus (MPa)	*Std. Dev.*
1	PP	38.16	0.15	3.28	0.03	1163.41	15.69
2	W10P90	34.13	0.45	1.46	0.13	2337.67	172.85
3	W10P70H20	29.24	0.21	1.18	0.13	2477.97	235.00
4	W10P60H30	27.16	0.81	0.94	0.09	2889.36	257.88
5	W10P50H40	27.22	0.65	0.96	0.10	2835.42	268.35
6	W10P40H50	24.78	0.64	0.83	0.05	2985.54	85.48
7	W10P30H60	22.65	0.48	0.87	0.06	2603.45	150.31
8	W20P80	39.89	0.74	1.62	0.04	2462.35	91.98
9	W20P60H20	28.96	0.68	1.22	0.06	2373.77	166.42
10	W20P50H30	27.45	0.84	0.96	0.11	2859.38	274.55
11	W20P40H40	25.87	0.29	0.92	0.03	2811.96	107.03
12	W20P30H50	23.78	0.64	0.84	0.05	2830.95	241.21
13	W20P20H60	23.11	0.86	0.76	0.08	3040.79	240.95

**Table 4:** UL 94 flame-retardant test result of wood-polymer composites.

No.	Sample Name	Flame Applying Time (s)	Burning Time (s)	UL 94 (Vertical) Rating
1	PP	10 s	30 s	Out of spec.
2	W10P90	10 s	30 s	Out of spec.
3	W10P70H20	10 s	30 s	Out of spec.
4	W10P60H30	10 s	30 s	Out of spec.
5	W10P50H40	10 s 30 s	No burning 38 s	V-1
6	W10P40H50	10 s 30 s	No burning 19 s	V-0
7	W10P30H60	10 s 30 s	No burning 7 s	V-0
8	W20P80	10 s	30 s	Out of spec.
9	W20P60H20	10 s	30 s	Out of spec.
10	W20P50H30	10 s 30 s	No burning 35 s	V-1
11	W20P40H40	10 s 30 s	No burning 35 s	V-1
12	W20P30H50	10 s 30 s	No burning 12 s	V-0
13	W20P20H60	10 s 30 s	No burning 3 s	V-0

## Data Availability

The original contributions presented in this study are included in the article. Further inquiries can be directed to the corresponding author.
